# EUPopLink Country report - Sweden

**DOI:** 10.12688/openreseurope.23451.2

**Published:** 2026-06-23

**Authors:** Zuhal Unalp Cepel, Sinem Abka

**Affiliations:** 1 Department of Political Science and International Relations, Dokuz Eylul Universitesi Isletme Fakultesi, Buca, İzmir, 35390, Turkey; 2 Department of Political Science and International Relations, Dokuz Eylul Universitesi Isletme Fakultesi, Buca, İzmir, 35390, Turkey

**Keywords:** Populism, Euroscepticism, Sweden

## Abstract

This paper examines the historical trajectory and contemporary transformation of Euroscepticism and populism within the Swedish political landscape. Sweden became a full member of the European Union (EU) in 1995. However, while traditional parties such as the Centre Party, the Liberal Party, and the Christian Democrats continue to advocate for European integration and supranational cooperation, the emergence of populist movements has significantly altered the national discourse.

The research highlights a critical shift from traditional, sovereignty-based Euroscepticism to a modern form of populism driven by neoliberal economic shifts, migration challenges, and domestic security concerns. A focal point of this analysis is the rise of the Sweden Democrats (SD), which entered parliament in 2010 and achieved a historic breakthrough in the 2022 general elections. As the second-largest party, the SD now exerts substantial influence over socio-economic and migration policies through the Tidö agreement with the governing coalition.

The analysis concludes that Sweden currently faces a dualistic future. First path is characterized by a “soft” Euroscepticism focused on environmental and human rights reforms, and the second path is defined by far-right extremism that challenges the fundamental principles of the EU. The transformative impact of the SD suggests that Swedish populism is no longer a peripheral phenomenon but a central force reshaping both national identity and EU affairs.

## Plain Language summary

Since joining the European Union in 1995, Sweden’s relationship with the bloc has been defined by a complex tug-of-war between international cooperation and national scepticism. Swedish politics is currently split between Europhile (pro-EU) and Eurosceptic (anti-EU or critical) forces.

The pro-EU Parties such as the Centre Party, Liberal Party, and Christian Democrats advocate for deeper integration, focusing on free trade, environmental standards, and collective security. However, the Eurosceptic and populist parties such as the Left Party and the Sweden Democrats (SD) have historically challenged the EU’s authority.

The most significant shift in Swedish politics occurred in the 2000s with the emergence of the SD. The party entered parliament in 2010 and became the second-largest political force in 2022. Unlike traditional Euroscepticism, which was often based on sovereignty, modern Swedish populism is driven by concerns over neoliberal economics, migration, and rising crime.

Today, Sweden stands at a crossroads. One path leads toward a “soft” Euroscepticism that seeks to reform the EU based on human rights and the environment. The other path, driven by far-right momentum, moves toward nationalistic policies that fundamentally challenge the EU’s founding principles. The SD’s current role as a key government supporter ensures that their transformative influence on migration and socio-economic policy will remain a central feature of Swedish and European affairs for years to come.

The present country report was prepared as part of the EUPopLink COST Action ( Andreadis & Vasilopoulou, 2014) following the EUPopLink Theoretical Framework and guidelines ( Lorimer *et al*., 2026).

## 1. Introduction

Sweden became a full member of the European Union (EU) in 1995 following a single-year negotiation period. However, it should be noted that the 1994 referendum for full membership resulted in 52.3% voting ‘yes’, 46.8% voting ‘no’, and 0.9% of blank votes ( Government Offices of Sweden, 2025). This suggests that a significant proportion of Swedish citizens held a negative view of full membership in the 1990s. In the 2000s, there are still Eurosceptic views in the Swedish society.

In the parliament, the Centre Party, the Christian Democrats, and the Liberal Party are Europhile parties. The Centre Party supports free trade, environmental policies, and international cooperation under the EU’s supranational authority. The Christian Democrats and the Liberals also support European integration policies involving closer cooperation. However, the Left Party and the SD have been Eurosceptic and populist political parties.

Growing populism in Sweden has had a negative impact on participation in European decision-making procedures. The participation of Swedes in EP elections decreased from 55.3% in 2019 to 50.7% in 2024. This trend can also be seen in the participation in the national elections as it decreased from 83.7% in 2018 to 80.2% in 2022 ( Fastesson Lindgren, 2024). Sweden’s populism took a different path to other European countries. The SD joined the parliament in 2010 and marked the beginning of populism in Swedish politics. While the rise of the SD is a notable feature of populism in Sweden, it could be argued that other parties, such as the Left Party, also exhibit populist tendencies.

In the latest general election, held in 2022, the SD received 20.5% of the vote, winning 73 seats as the second biggest party in parliament ( Güngör, 2023; PolitPro, n.d). This is regarded as a turning point in Sweden’s history as a tolerant country. The party has a confidence-and-supply agreement with the government despite not being part of the coalition. It has been awarded four committee chairmanships in recognition of its external support ( Güngör, 2023; Statista, 2025).

## 2. Populist political actors

Populism, in its most general definition, is a political ideology that establishes an opposition between ‘the people’ and ‘the elites’ and claims to represent the interests of the ordinary people. In the context of this definition, it is observed that some parties in Sweden use populist discourses to varying degrees.

The Swedish parliament has 349 members and eight political parties in the 2022–2026 term. The conservative parties include the Sweden Democrats (SD), the Christian Democrats, the Moderate Party and the Liberal Party, while the leftists are the Social Democratic Party, the Green Party, the Centre Party and the Left Party ( Sveriges Riksdag, n.d.). Out of these parties, the Left Party is left-wing, populist and Eurosceptic, and opposes economic inequalities and elites. It portrays economic inequality as a struggle between elites and workers, arguing that the EU favours financial interests over democracy and welfare. SD, on the other hand has been the most influential far-right and nativist political party in Sweden. Its origins depend on the Nazi movement, and it has a nationalist, antisemitic and anti-immigrant stance. This can be understood from the fact that some founding members belong to a white supremacist group namely ‘Keep Sweden Swedish’, and a couple of those members had close links with Nazi organizations serving the ‘Waffen SS’ of Hitler’s military units ( The Standard, 2022). While some of these members explicitly advocated anti-Islamism and antisemitism, others even attended party meetings in Nazi uniforms until the mid-1990s. Populist rhetoric is also used by other mainstream parties, but this does not make their beliefs popülist ( Gülal, 2021). Accordingly, this paper focuses mainly on SD and the Left Party when analyzing the phenomenon of Euroscepticism-populism nexus in Swedish politics.

In 2005, Jimmie Åkesson, the head of the Sweden Democratic Youth, took over as leader of the SD. Previously, Åkesson was a web developer and a member of the Moderate Party. He redesigned the party’s image following the removal of several neo-Nazi figures from the party logo to influence new voters. Consequently, the party advanced to the next stage of transformation between 2006 and 2010. The years between 2012 and 2015 reflected a change in the party’s approach to racism, with the slogan of “zero tolerance to racism” ( Güngör, 2023). During the global financial crisis, the party adopted a populist stance supporting ‘ordinary people’ against ‘corrupt elite’. This brought major media attention and their first electoral success in 2010 elections with gaining 20 seats in the parliament ( Tomson, 2020). It became the second biggest party in the parliament gaining the power to influence the political agenda by supporting the right-wing coalition government from outside.

Following the changes in party structure, the party has gained supporters from young people, the unemployed, and the students ( Halhallı, 2024). Apparently, it expanded its voter base with the discourse of protecting Sweden’s cultural homogeneity and the welfare state as the party members represent themselves as the supporters of Swedish culture, opposing the migration and integration policies ( Janssen, 2023). However, their party programs and election manifestos do not include any evidence of classical racism; rather, they shape their views on national belonging through culture. In this respect, it seems to follow a left-wing policy on welfare expenditures and a right-wing policy on traditional values such as morality and identity ( Dutceac Segesten, 2018). There have been documented instances of party members making exclusionist statements; for example, some members issued an invitation to celebrate the 1939 Nazi invasion of Poland, a move widely regarded as an endorsement of extremist ideologies ( Gera, 2022).

The SD places a strong emphasis on four essential components: Prosperity, a united nation, a safe society, and a strict immigration policy. Islam is perceived as an extremist religion, and migrants are linked to problems like organized crime, terrorism, and human trafficking ( Mulinari & Neergaard, 2014). SD’s populist discourse, however, has been shaped by two main points: The increasing influx of migrants and the increasing crime rates. These approaches make SD the closest party to the authoritarian pole in Sweden ( Tezcan, 2022). On the other hand, the Left Party was founded in 1917 with an ideology that was close to Soviet-style communism. In the 1980s, references to communism were removed from the party’s name and program ( Radio Sweden, 2014). It has followed a strict anti-EU approach until 1995 and has gained more votes than the other Swedish political parties in the latest national and European elections.

The SD and the Left Party have two diverse populist approaches in terms of representing the electorate. Although the SD’s party programs and election manifestos cover issues such as unemployment, taxation, and public expenditure, the party’s main focus remains on the intersection between migration and the rising crime rate in Sweden. The influx of immigrants has caused serious tensions within Swedish society, spotlighting the country’s national sentiments. SD’s populist announcement to the public is, “We say what you think”. Accordingly, the party argues that non-Western refugees, especially Muslims, damage secular Sweden ( Tomson, 2020). It states that the reason for the rise in crime rates and gang shootings is the migrants. The party aims to lower the number of immigrants to zero and proposes to establish “Swedish Centres” in the vulnerable regions of the country ( Halhallı, 2024).

The Left Party, on the other side, stresses the rights of the working class and the protection of Sweden’s social democratic background. It promotes a humanitarian and solidarity-based approach to immigration, grounded in respect for international conventions and the right to asylum. The party argues that Sweden has both the moral and practical capacity to offer refuge to people fleeing war and persecution. It supports permanent residence permits and emphasizes the importance of family reunification and dignified, legally secure asylum procedures. Effective integration, according to the party, depends on a strong welfare state that provides equal access to education, language training, and employment opportunities, thereby preventing exploitation and social marginalization ( Vänsterpartiet, 2024).

In addressing rising crime rates, the Left Party rejects punitive approaches in favour of prevention and social inclusion. It emphasizes adequate resources for police, social services, and local communities, but argues that sustainable safety arises from reducing inequality and strengthening social cohesion. The party views crime primarily as a consequence of social and economic exclusion, advocating policies that promote stable communities, meaningful employment, and gender equality. It also draws attention to Palestinian rights through political pressure and sanctions against Israel, criticizing the EU’s silence ( Hörnquist, 2024). The party opposes Sweden’s NATO membership, but supported the formation of a new pro-Ukrainian European left party in 2024. This suggests a shift in its support from pro-European voters, as shown by the 2024 EP elections ( Elvander, 2025).

## 3. Positions on ‘Europe’ and the populism-euroscepticism nexus

Euroscepticism is a strategy employed by populist parties to attract voters by blaming the EU for crises. Their propaganda against joining the European Monetary Union convinced Swedish society to vote against it in the 2003 referendum. The SD and the Left Party followed Eurosceptical political approaches until the 2000s. Their Euroscepticism and populism differ significantly in terms of discourses and the fields they criticize. Concurrently, these two political parties have different approaches to human rights, equality, solidarity and democratic values.

Since 1995, the Left Party had been highly critical of the EU for prioritizing market liberalization, privatization, and fiscal austerity over social justice, workers’ rights, and welfare. The party suggests that rather than the technocratic dominance within the EU, national parliaments and democratic institutions should make economic decisions to protect equality and public welfare. However, in the 2000s, this stance evolved into the promotion of EU values, such as protecting the rights of minorities and refugees in Europe. This approach has increased support for leftist ideology and the Left Party in the EP ( Dudzińska, 2024). The party has kept populist promises on European integration, recently rejecting EU policy on climate and social issues. It argues that EU neoliberal policies harm the welfare state by decreasing social rights and weakening national sovereignty ( Hörnquist, 2024).

For SD, the EU represents a threat to national self-determination and cultural identity, rather than a community of shared governance. While the party supports free trade and free movement within Europe, it rejects what it perceives as the EU’s intrusion into domestic affairs and efforts toward deeper political integration. The party supported Swexit, but the offer has now been withdrawn ( Gülal, 2021). Contrary to this move, the SD leader Åkesson, currently describes the EU as the “source of evil” and does not support the EU’s condemnation of Hungary for violating the rule of law ( Kenes, 2021). Åkesson is critical of Germany’s “dominant role” in shaping the EU policies which fail to stop migration ( Ateş, 2023). According to Riegert, Åkesson’s discourse makes the party one of the natural allies of other populist and far right parties in Europe. The party has already been against migration before the 2015 refugee crisis as migration has been perceived as the biggest external threat to Sweden since the Second World War ( Riegert, 2023). In the 2022 election campaign, the party promised to introduce the strictest migration law to make Sweden “safe again” ( Güngör, 2023). Consequently, the government signed the Tidö Agreement with the SD due to its rising popularity in Swedish society. The agreement has enabled the SD to achieve its goals, such as setting minimum standards for asylum policies, tightening the conditions for family reunification, and encouraging the return of refugees.

In terms of representation in the EP, the SD is one of the Eurosceptic party groups, the Group of European Conservatives and Reformists (ECR). The party criticizes multiculturalism and promotes the protection of national identity and closer cooperation with Nordic countries. It also opposes deeper integration in the EU ( Güngör, 2023). Unlike SD, the Left Party is represented in the EP under the Left Group-GUE/NGL, which aims to protect workers, the environment, women’s rights, peace and human rights. It is not totally against the EU; however, it rejects EU austerity measures, privatization to the benefit of big businesses and militarization. It supports transparency and accountability to stop the rise of the far-right in Europe and to protect civil liberties ( The Left in the European Parliament, n.d.)

In conclusion, unlike the Left Party, which has moved away from anti-EU positioning due to its EP involvement, the SD remains committed to hard Euroscepticism. This enables the party to maintain its radical-populist appeal and pursue an ethnocentric agenda rooted in nativist principles.

## 4. Responses to Euroscepticism and consequences

The SD and the Left Party displayed a similar approach to European integration until the 2000s. The Left Party opposed the EU because it was representing the “Western imperialism”. It supported leaving the EU, with “Swexit” in its party program, citing “unelected bureaucrats in Brussels” making decisions for Europe. However, the party’s anti-EU stance changed in the 2000s, and the demand for Swexit was silenced under the leadership of the party leader, Jonas Sjöstedt. This change was explained by the fact that they were not aligned with the far-right SD. Ironically, a few months later SD also changed its approach, abandoning the Swexit discussion. The geopolitical environment in the 2000s led to a shift in the Swedish parliament, leaving no voice for Euroscepticism among both the mainstream and populist parties ( Elvander, 2025).

The Left Party’s anti-EU promises attracted mostly the labour movement in the north of the country, and the party increased its vote share from 10.3 to 23.3% in 2022. Unlike the Christian Democrats, anti-Israeli discourses and calls for solidarity for the Palestinians impacted the European voters in the 2024 European Parliament (EP) elections. This resulted in an increase in vote share from 6.8 to 11.06% in the EP ( Hörnquist, 2024). Nevertheless, in the 2024 national elections, the Moderate Party, the Christian Democrats and the Liberal Party reached a compromise on SD’s request to sign the Tidö Agreement, which set out policies to address rising crime rates and migration. This was an important step in gaining external support from SD for the formation of a new coalition government. However, the Tidö Agreement has been criticized for undermining human rights and the principle of equality before the law.

## 5. Short conclusion

Euroscepticism is an old phenomenon in Swedish politics, defended by both right-wing and left-wing parties. Populism, on the other hand, is a new phenomenon that began in the 2000s due to the effects of neoliberal economic policies, rising numbers of migrants, and crime levels in the country. This has empowered far-right movements in Sweden, such as the SD, which have successfully persuaded the Swedish population with populist discourses on economic, migration, and security policies. On the other hand, the Left Party is becoming more popular among European citizens because of its promises regarding environmental protection and human rights. However, the SD has a direct impact on Sweden’s traditional socio-economic and migration policies because of its success in national elections. It is clear that the SD, along with the other populist far-right parties in EU countries, has a huge transformative impact not only on national politics but also on EU affairs. For this reason, Sweden faces two potential paths for future politics. One path involves a softer form of Euroscepticism that prioritizes meeting the demands of the European public on environmental, economic and human rights issues. The other path involves far-right extremism, which is far removed from the EU’s founding principles.

## Ethics and consent

Ethical approval and consent were not required for this paper.

## Data Availability

No new data were created or analyzed in this study. Data sharing is not applicable to this paper.
