# Supplementary material for: EUPopLink Country report - Sweden
Source: Open Res Eur. 2026 Jun 23;6:120. Originally published 2026 Apr 16. [Version 2] doi: 10.12688/openreseurope.23451.2 (PMC13309770; doi:10.12688/openreseurope.23451.2)
Supplement: Peer review history

# Reviewer report: Reviewer response for version 2

**DOI:** 10.21956/openreseurope.26249.r76356

**Published:** 2026-06-26

**Authors:** Michael A Hansen

**Affiliations:** 1 University of Turku, Turku, Finland

The authors have usefully revised the report, dropping the strongest unsupported claim, restructuring to match the collection, and reasonably grounding definitional scope and literature coverage in the shared EUPopLink framework. Other responses address the form of my concerns rather than their substance: the exclusionary-rhetoric claim is relabelled but not evidenced, and the systematic demonstration of Euroscepticism and over-reliance on a single figure's rhetoric remain open. Indexable as a short country report, with those points left for readers to weigh.

Is the case presented with sufficient detail to be useful for teaching or other practitioners?

No

Is the work clearly and accurately presented and does it cite the current literature?

No

If applicable, is the statistical analysis and its interpretation appropriate?

Not applicable

Are all the source data underlying the results available to ensure full reproducibility?

No source data required

Are the conclusions drawn adequately supported by the results?

Partly

Is the background of the case’s history and progression described in sufficient detail?

Partly

Reviewer Expertise:

I have published on far-right and radical right wing populist parties, including a book and approximately a dozen articles.

I confirm that I have read this submission and believe that I have an appropriate level of expertise to confirm that it is of an acceptable scientific standard.

# Reviewer report: Reviewer response for version 2

**DOI:** 10.21956/openreseurope.26249.r76357

**Published:** 2026-06-26

**Authors:** Greg Simons

**Affiliations:** 1 Daffodil International University, Dhaka, Bangladesh

Noticeable improvements have been made to the work in the revision process.

Is the case presented with sufficient detail to be useful for teaching or other practitioners?

Partly

Is the work clearly and accurately presented and does it cite the current literature?

Partly

If applicable, is the statistical analysis and its interpretation appropriate?

Not applicable

Are all the source data underlying the results available to ensure full reproducibility?

Yes

Are the conclusions drawn adequately supported by the results?

Partly

Is the background of the case’s history and progression described in sufficient detail?

Partly

Reviewer Expertise:

international relations, political marketing, politics, mass communication, journalism

I confirm that I have read this submission and believe that I have an appropriate level of expertise to confirm that it is of an acceptable scientific standard.

# Reviewer report: Reviewer response for version 1

**DOI:** 10.21956/openreseurope.25378.r73191

**Published:** 2026-04-30

**Authors:** Greg Simons

**Affiliations:** 1 Daffodil International University, Dhaka, Bangladesh

This present article seeks to address the rise of populism in Sweden, and how populism manifests differently along the traditional political spectrum of left versus right wing politics. In general, this is a reasonably accurate picture but misses some important nuances. 

 1) Along Sweden has long preached tolerance to the rest of the world, it has not really practiced tolerance in good faith. Those who 'violate' the 'sacred' corridor of opinion are castigated and isolated from society. Sweden relief and works on consensus, which is often done through coercion rather than a genuine conformity. 

 2) The issue of the public referendum on the vote for adopting the Euro as national currency to replace the Swedish Crown was missed. It is a good indicator of the latent and underlying Swedish nationalism and Euroscepticism. Especially when the Yes Vote lobby attempted to use the murder of Anna Lind (foreign minister) to prime and mobilise and yes vote, and they failed. 

 3) A discussion on the political construct of Sweden as a humanitarian superpower should be discussed, which was an attempt to popularise miss migration, and not by the left. Further concepts should also be explored, such as the now abandoned by the mainstream establishment political parties of the idea of Swedish exceptionalism and this arrogant and didactic approach to the rest of the world. 

 4) SD have certainly normalised this traditionalist and nationalist discourse, the breaking of the opinion corridor, which occurred as a result of the chaotic and deeply mismanaged results of mass migration after 2015. This chaos and destruction of the Swedish self-image contributed to a nostalgia of an idealised concept and memory of a 'pure' Sweden from a cultural and identity perspective. 

 5) SD definitely have a non-inclusive approach to policy articulation and a hardened law and order approach. However, there are many voters from the immigrant communities, including Muslims, that vote for them because they want law and order, which the traditional parties have not managed at all. Given 1 in 5 people in Sweden are foreign born or born to parents that are foreign born, it is a large, but a very heterogeneous community. 

 6) I would not characterise SD as being Nazi in orientation, but rather demagogic and opportunistic. Left Party is definitely with some Soviet nostalgia, around 10 years ago the then party leader used the economic policy platform of praising Stalin and proposing the introduction of a 5-year plan.

Is the case presented with sufficient detail to be useful for teaching or other practitioners?

Partly

Is the work clearly and accurately presented and does it cite the current literature?

Partly

If applicable, is the statistical analysis and its interpretation appropriate?

Not applicable

Are all the source data underlying the results available to ensure full reproducibility?

Yes

Are the conclusions drawn adequately supported by the results?

Partly

Is the background of the case’s history and progression described in sufficient detail?

Partly

Reviewer Expertise:

international relations, political marketing, politics, mass communication, journalism

I confirm that I have read this submission and believe that I have an appropriate level of expertise to confirm that it is of an acceptable scientific standard, however I have significant reservations, as outlined above.

# Response

**Published:** 2026-05-12

**Authors:** Zuhal Unalp-Cepel

The authors appreciate the constructive feedback and the opportunity to refine this manuscript.

 1. We would like to clarify that our report does not depart from a normative assumption of Sweden as an inherently tolerant nation; rather, it seeks to objectively map the current populism-euroscepticism nexus. While the internal pressures for political conformity are a significant subject of study, incorporating a detailed critique of Swedish social coercion would diverge from the specific focus on party-level rhetoric.

 2. We agree that the 2003 referendum is a crucial indicator of the underlying Euroscepticism in Sweden. In response to the reviewer’s suggestion, we have updated the manuscript to highlight this significant event. We have specifically noted how populist rhetoric and propaganda against joining the European Monetary Union played a decisive role in convincing the Swedish public to vote against the adoption of the Euro. While the tragic murder of Anna Lindh is a noteworthy historical detail, we have focused on the broader strategic implications for Euroscepticism to remain consistent with the report’s concise analytical scope.

 3. The reviewer raises an interesting point regarding the 'humanitarian superpower' narrative and the concept of Swedish exceptionalism. While these are significant elements of the Swedish political discourse, providing a thorough analysis of these constructs would exceed the 3,000-word limit and shift the report's primary focus. Our analysis is strictly bound by the common framework of this Special Issue, which prioritizes the nexus between populism and Euroscepticism. Within this scope, migration is addressed as a contextual factor rather than a central theoretical theme.

 4. We have revised the text to more clearly indicate how the influx of immigrants exacerbated social tensions and brought national sentiments to the forefront. By highlighting these tensions, the report acknowledges the shift in the Swedish self-image and the subsequent normalization of traditionalist narratives by the SD.

 5. We thank the reviewer for highlighting the heterogeneity of the immigrant electorate in Sweden and the nuanced reasons behind their voting behaviour, such as the focus on 'law and order.' While this is a compelling sociological observation, incorporating a detailed analysis of voter demographics and motivations would deviate from our report’s primary objective: mapping the populism-euroscepticism nexus at the party level. Given the strict 3,000-word constraint, we have prioritized the party's official positions and rhetoric. We believe that while the immigrant vote is an important phenomenon, it does not alter the core analysis of the SD's populist-eurosceptic framework as defined by the Editorial Board.

 6. The reviewer’s point regarding the nuances of party labelling is well-taken. Regarding the SD, our characterization relies on the predominant scholarly literature and investigative reports that document the party's ideological roots. However, to remain objective and avoid reductive labelling, we have ensured the text focuses on their documented political actions and rhetoric rather than relying solely on static labels.

# Response

**Published:** 2026-06-04

**Authors:** Zuhal Unalp-Cepel

1. **Along Sweden has long preached tolerance to the rest of the world, it has not really practiced tolerance in good faith. Those who 'violate' the 'sacred' corridor of opinion are castigated and isolated from society. Sweden relief and works on consensus, which is often done through coercion rather than a genuine conformity.**

**Response:** We would like to clarify that our report does not depart from a normative assumption of Sweden as an inherently tolerant nation; rather, it seeks to objectively map the current populism-euroscepticism nexus. While the internal pressures for political conformity are a significant subject of study, incorporating a detailed critique of Swedish social coercion would diverge from the specific focus on party-level rhetoric.

1. **The issue of the public referendum on the vote for adopting the Euro as national currency to replace the Swedish Crown was missed. It is a good indicator of the latent and underlying Swedish nationalism and Euroscepticism. Especially when the Yes Vote lobby attempted to use the murder of Anna Lind (foreign minister) to prime and mobilise and yes vote, and they failed.**

**Response:** We agree that the 2003 referendum is a crucial indicator of the underlying Euroscepticism in Sweden. In response to the reviewer’s suggestion, we have updated the manuscript to highlight this significant event. We have specifically noted how populist rhetoric and propaganda against joining the European Monetary Union played a decisive role in convincing the Swedish public to vote against the adoption of the Euro. While the tragic murder of Anna Lindh is a noteworthy historical detail, we have focused on the broader strategic implications for Euroscepticism to remain consistent with the report’s concise analytical scope.

1. **A discussion on the political construct of Sweden as a humanitarian superpower should be discussed, which was an attempt to popularise miss migration, and not by the left. Further concepts should also be explored, such as the now abandoned by the mainstream establishment political parties of the idea of Swedish exceptionalism and this arrogant and didactic approach to the rest of the world.**

**Response:** The reviewer raises an interesting point regarding the 'humanitarian superpower' narrative and the concept of Swedish exceptionalism. While these are significant elements of the Swedish political discourse, providing a thorough analysis of these constructs would exceed the 3,000-word limit and shift the report's primary focus. Our analysis is strictly bound by the common framework of this Special Issue, which prioritizes the nexus between populism and Euroscepticism. Within this scope, migration is addressed as a contextual factor rather than a central theoretical theme.

1. **SD have certainly normalised this traditionalist and nationalist discourse, the breaking of the opinion corridor, which occurred as a result of the chaotic and deeply mismanaged results of mass migration after 2015. This chaos and destruction of the Swedish self-image contributed to a nostalgia of an idealised concept and memory of a 'pure' Sweden from a cultural and identity perspective.**

**Response:** We have revised the text to more clearly indicate how the influx of immigrants exacerbated social tensions and brought national sentiments to the forefront. By highlighting these tensions, the report acknowledges the shift in the Swedish self-image and the subsequent normalization of traditionalist narratives by the SD.

1. **SD definitely have a non-inclusive approach to policy articulation and a hardened law and order approach. However, there are many voters from the immigrant communities, including Muslims, that vote for them because they want law and order, which the traditional parties have not managed at all. Given 1 in 5 people in Sweden are foreign born or born to parents that are foreign born, it is a large, but a very heterogeneous community.**

**Response:** We thank the reviewer for highlighting the heterogeneity of the immigrant electorate in Sweden and the nuanced reasons behind their voting behaviour, such as the focus on 'law and order.' While this is a compelling sociological observation, incorporating a detailed analysis of voter demographics and motivations would deviate from our report’s primary objective: mapping the populism-euroscepticism nexus at the party level. Given the strict 3,000-word constraint, we have prioritized the party's official positions and rhetoric. We believe that while the immigrant vote is an important phenomenon, it does not alter the core analysis of the SD's populist-eurosceptic framework as defined by the Editorial Board.

1. **I would not characterise SD as being Nazi in orientation, but rather demagogic and opportunistic. Left Party is definitely with some Soviet nostalgia, around 10 years ago the then party leader used the economic policy platform of praising Stalin and proposing the introduction of a 5-year plan.**

**Response:** The reviewer’s point regarding the nuances of party labelling is well-taken. Regarding the SD, our characterization relies on the predominant scholarly literature and investigative reports that document the party's ideological roots. However, to remain objective and avoid reductive labelling, we have ensured the text focuses on their documented political actions and rhetoric rather than relying solely on static labels.

1. **Request by the Editorial Board on 21 May 2026: “Rename the sections to align with the structure used in the other country reports (you can consult examples here:** https://open-research-europe.ec.europa.eu/collections/eupoplink **). In addition to the Introduction, the sections should be structured as follows:**
  1. **Populist political actors**
  2. **Positions on ‘Europe’ and the Populism–Euroscepticism nexus**
  3. **Responses to Euroscepticism and consequences**
  4. **Short conclusions”**

**“This should be relatively straightforward to carry out in two steps:**

1. **Delete and integrate (and possibly slightly reduce) the section “Main political discourses of SD and left party” into Section 2 (“Populist   political actors”);**
2. **Add Section 4 (“Responses to Euroscepticism and consequences”), referring to the reactions of mainstream parties, although there is no need for excessive detail—you may also consult other country reports as a guide.”**

**Response: **The titles have been revised in accordance with the editors’ recommendations and aligned with the format used in the sample country reports available at the provided link. In addition, the citation system has been updated to match the style used in those reports. All modifications can be reviewed in the main text using the “Track Changes” feature.

# Reviewer report: Reviewer response for version 1

**DOI:** 10.21956/openreseurope.25378.r72548

**Published:** 2026-04-22

**Authors:** Michael A Hansen

**Affiliations:** 1 University of Turku, Turku, Finland

Summary: This report examines the development of Euroscepticism and populism in Sweden, with particular attention to the role of the Sweden Democrats (SD). It outlines Sweden’s historical relationship with the European Union and highlights recent political shifts, especially the growing influence of populist actors in national and European politics. While the report provides useful descriptive context and traces key developments, its analytical contribution remains unclear, and it does not consistently engage with relevant academic literature.

 Main Critique:  

 -The report does not read as a systematic historical and contemporary analysis but instead at times reads more as an editorial narrative than a systematic analysis, with several claims not clearly supported by evidence. The lack of a clearly articulated analytical framework and limited engagement with existing scholarship make it difficult to assess the strength and originality of its contributions.

 Introduction:

 -The introduction leaves the central thesis of the report ambiguous. While several descriptive statistics are presented, the overarching argument and analytical focus are not clearly articulated.

 -Strong assertions, such as "no other party is as clearly and unambiguously defined as a populist party as the SD." should be supported with empirical evidence or appropriate references.

 Political parties and populism: 

 -The report defines populism as follows: “Populism, in its most general definition, is a political ideology that establishes an opposition between ‘the people’ and ‘the elites’ and claims to represent the interests of the ordinary people.” Yet, it does not engage with the extensive scholarly debates on defining populism, which limits its ability to situate this definition within existing research.

 -The statement “However, the party members occasionally do not hesitate to make racist speeches” requires further clarification and support. The claim is not accompanied by citations or examples, making it difficult to evaluate. It is unclear whether this reflects current party behavior, isolated incidents, or a broader pattern. The phrasing also risks overgeneralization and would benefit from more precise wording and evidence.

 Analysis:

 -While the report asserts that the Sweden Democrats convey Eurosceptic positions, this claim is not systematically demonstrated. The analysis places considerable emphasis on Jimmie Åkesson, at times treating his rhetoric as representative of the party as a whole, while also relying heavily on secondary interpretations of his positions. A more systematic assessment - such as analysis of party manifestos, speeches, or voting behavior - would strengthen the argument.

 Literature:

 -The report engages only minimally with the academic literature on far-right populist parties, which makes it difficult to assess how its arguments relate to, extend, or challenge existing scholarship. A more thorough integration of relevant research would help clarify the report’s theoretical positioning and contribution.

Is the case presented with sufficient detail to be useful for teaching or other practitioners?

No

Is the work clearly and accurately presented and does it cite the current literature?

No

If applicable, is the statistical analysis and its interpretation appropriate?

Not applicable

Are all the source data underlying the results available to ensure full reproducibility?

No source data required

Are the conclusions drawn adequately supported by the results?

Partly

Is the background of the case’s history and progression described in sufficient detail?

Partly

Reviewer Expertise:

I have published on far-right and radical right wing populist parties, including a book and approximately a dozen articles.

I confirm that I have read this submission and believe that I have an appropriate level of expertise to state that I do not consider it to be of an acceptable scientific standard, for reasons outlined above.

# Response

**Published:** 2026-05-12

**Authors:** Zuhal Unalp-Cepel

The authors appreciate the feedback and the opportunity to refine this manuscript.

 1. We acknowledge the reviewer’s perspective on the report's narrative style. However, it is important to clarify that this manuscript was authored as a country report under the specific guidelines and 3,000-word limit set by the Editorial Board. To ensure analytical rigor within these constraints, we utilized the framework by Andreadis and Vasilopoulou (2026). The systematic nature of this analysis is underpinned by the EUPopLink Theoretical Framework, as explicitly stated in the Plain Language Summary of the report.

 2. The authors acknowledge the importance of situating the analysis within the broader academic literature. However, as noted previously, the theoretical positioning and the relevant scholarly debates for this Special Issue were pre-defined by the Editorial Board in the project’s overarching framework (Andreadis & Vasilopoulou, 2026). Our primary task was to apply this shared framework to the Swedish case within a strict 3,000-word limit. Consequently, we focused on providing a data-driven country analysis rather than duplicating the theoretical discussions already presented in the collection’s opening chapters.

 3. We acknowledge that populism is a contested concept with a vast scholarly literature. However, as this is a country report designed for a specific Special Issue, we have intentionally adopted the overarching definition and framework established for this collection to ensure comparative consistency. This framework is detailed in the introductory chapter by Andreadis and Vasilopoulou (2026). Given the strict length constraints, we focused on applying this shared conceptual lens to the Sweden country report case rather than engaging in broad definitional debates.

 4. We agree with the reviewer that the term "racist" might carry a judgmental tone that exceeds the boundaries of academic neutrality. Consequently, we have replaced it with "exclusionist statements" and "radical right-wing rhetoric" to ensure a more objective and descriptive analysis. This change maintains the critical assessment of the party's actions while adhering to a more formal academic register. We also believe the report already provides the necessary evidentiary basis for this statement by citing documented instances, such as the references to the Nazi invasion of Poland (Gera, 2022) and the party’s historical links to white supremacist movements (The Standard, 2022).

 5. While we agree that a comprehensive analysis of manifestos and voting behaviour would be valuable for a full-length research article, such an undertaking is beyond the feasible scope of a 3,000-word country report. Within these limits, we focused on the leadership's rhetoric—specifically Jimmie Åkesson’s—as it serves as a primary and highly influential indicator of the party's strategic direction. This approach aligns with the editorial goal of mapping the 'populism-euroscepticism nexus' in a concise manner. We believe that focusing on these key rhetorical positions sufficiently demonstrates the party's stance within the framework provided for this Special Issue.

 6.The authors acknowledge the importance of situating the analysis within the broader academic literature. However, as noted previously, the theoretical positioning and the relevant scholarly debates for this Special Issue were pre-defined by the Editorial Board in the project’s overarching framework (Andreadis & Vasilopoulou, 2026). Our primary task was to apply this shared framework to the Swedish case within a strict 3,000-word limit. Consequently, we focused on providing a data-driven country analysis rather than duplicating the theoretical discussions already presented in the collection’s opening chapters.

# Response

**Published:** 2026-06-04

**Authors:** Zuhal Unalp-Cepel

1. **The report does not read as a systematic historical and contemporary analysis but instead at times reads more as an editorial narrative than a systematic analysis, with several claims not clearly supported by evidence. The lack of a clearly articulated analytical framework and limited engagement with existing scholarship make it difficult to assess the strength and originality of its contributions.**

**Response:** We acknowledge the reviewer’s perspective on the report's narrative style. However, it is important to clarify that this manuscript was authored as a country report under the specific guidelines and 3,000-word limit set by the Editorial Board. To ensure analytical rigor within these constraints, we utilized the framework by Andreadis and Vasilopoulou (2026). The systematic nature of this analysis is underpinned by the EUPopLink Theoretical Framework, as explicitly stated in the Plain Language Summary of the report.

1. **Introduction: The introduction leaves the central thesis of the report ambiguous. While several descriptive statistics are presented, the overarching argument and analytical focus are not clearly articulated. & Strong assertions, such as "no other party is as clearly and unambiguously defined as a populist party as the SD." should be supported with empirical evidence or appropriate references.**

**Response: **The central thesis of the report is explained in EUPopLink Theoretical Framework and Guidelines as stated in the Plain Language Summary of the report. Regarding the characterization of the SD, while we stand by the observation, we have removed the assertive phrasing to maintain a more balanced tone suitable for this report’s scope. This change ensures our analysis remains focused on the broader findings without requiring extensive empirical detours that the word count cannot accommodate.

1. **Political parties and populism: The report defines populism as follows: “Populism, in its most general definition, is a political ideology that establishes an opposition between ‘the people’ and ‘the elites’ and claims to represent the interests of the ordinary people.” Yet, it does not engage with the extensive scholarly debates on defining populism, which limits its ability to situate this definition within existing research.**

**Response: **We acknowledge that populism is a contested concept with a vast scholarly literature. However, as this is a country report designed for a specific Special Issue, we have intentionally adopted the overarching definition and framework established for this collection to ensure comparative consistency. This framework is detailed in the introductory chapter by Andreadis and Vasilopoulou (2026). Given the strict length constraints, we focused on applying this shared conceptual lens to the Sweden country report case rather than engaging in broad definitional debates.

1. **The statement “However, the party members occasionally do not hesitate to make racist speeches” requires further clarification and support. The claim is not accompanied by citations or examples, making it difficult to evaluate. It is unclear whether this reflects current party behaviour, isolated incidents, or a broader pattern. The phrasing also risks overgeneralization and would benefit from more precise wording and evidence.**

**Response:** We agree with the reviewer that the term "racist" might carry a judgmental tone that exceeds the boundaries of academic neutrality. Consequently, we have replaced it with "exclusionist statements" and "radical right-wing rhetoric" to ensure a more objective and descriptive analysis. This change maintains the critical assessment of the party's actions while adhering to a more formal academic register. We also believe the report already provides the necessary evidentiary basis for this statement by citing documented instances, such as the references to the Nazi invasion of Poland (Gera, 2022) and the party’s historical links to white supremacist movements (The Standard, 2022).

1. **Analysis: While the report asserts that the Sweden Democrats convey Eurosceptic positions, this claim is not systematically demonstrated. The analysis places considerable emphasis on Jimmie Åkesson, at times treating his rhetoric as representative of the party as a whole, while also relying heavily on secondary interpretations of his positions. A more systematic assessment - such as analysis of party manifestos, speeches, or voting behaviour - would strengthen the argument.**

**Response:** While we agree that a comprehensive analysis of manifestos and voting behaviour would be valuable for a full-length research article, such an undertaking is beyond the feasible scope of a 3,000-word country report. Within these limits, we focused on the leadership's rhetoric—specifically Jimmie Åkesson’s—as it serves as a primary and highly influential indicator of the party's strategic direction. This approach aligns with the editorial goal of mapping the 'populism-euroscepticism nexus' in a concise manner. We believe that focusing on these key rhetorical positions sufficiently demonstrates the party's stance within the framework provided for this Special Issue.

1. **Literature: The report engages only minimally with the academic literature on far-right populist parties, which makes it difficult to assess how its arguments relate to, extend, or challenge existing scholarship. A more thorough integration of relevant research would help clarify the report’s theoretical positioning and contribution.**

**Response:** The authors acknowledge the importance of situating the analysis within the broader academic literature. However, as noted previously, the theoretical positioning and the relevant scholarly debates for this Special Issue were pre-defined by the Editorial Board in the project’s overarching framework (Andreadis & Vasilopoulou, 2026). Our primary task was to apply this shared framework to the Swedish case within a strict 3,000-word limit. Consequently, we focused on providing a data-driven country analysis rather than duplicating the theoretical discussions already presented in the collection’s opening chapters.

1. **Request by the Editorial Board on 21 May 2026: “Rename the sections to align with the structure used in the other country reports (you can consult examples here:** https://open-research-europe.ec.europa.eu/collections/eupoplink **). In addition to the Introduction, the sections should be structured as follows:**
  1. **Populist political actors**
  2. **Positions on ‘Europe’ and the Populism–Euroscepticism nexus**
  3. **Responses to Euroscepticism and consequences**
  4. **Short conclusions”**

**“This should be relatively straightforward to carry out in two steps:**

1. **Delete and integrate (and possibly slightly reduce) the section “Main political discourses of SD and left party” into Section 2 (“Populist   political actors”);**
2. **Add Section 4 (“Responses to Euroscepticism and consequences”), referring to the reactions of mainstream parties, although there is no need for excessive detail—you may also consult other country reports as a guide.”**

**Response: **The title has been revised in accordance with the editors’ recommendations and aligned with the format used in the sample country reports available at the provided link. In addition, the citation system has been updated to match the style used in those reports. All modifications can be reviewed in the main text using the “Track Changes” feature.
